# The Epidemiology of Sarcoma

**DOI:** 10.1186/2045-3329-2-14

**Published:** 2012-10-04

**Authors:** Zachary Burningham, Mia Hashibe, Logan Spector, Joshua D Schiffman

**Affiliations:** 1 Department of Family And Preventive Medicine, University of Utah, 2000 Circle of Hope, HCI-4245, Salt Lake City, UT, 84112, USA; 2 Division of Epidemiology/Clinical Research, Department of Pediatrics and Masonic Cancer Center, University of Minnesota, Minneapolis, MN, USA; 3 Division of Pediatric Hematology/Oncology, Center for Children's Cancer Research, Huntsman Cancer Institute, University of Utah, Salt Lake City, UT, USA

## Abstract

Sarcomas account for over 20% of all pediatric solid malignant cancers and less than 1% of all adult solid malignant cancers. The vast majority of diagnosed sarcomas will be soft tissue sarcomas, while malignant bone tumors make up just over 10% of sarcomas. The risks for sarcoma are not well-understood. We evaluated the existing literature on the epidemiology and etiology of sarcoma. Risks for sarcoma development can be divided into environmental exposures, genetic susceptibility, and an interaction between the two. HIV-positive individuals are at an increased risk for Kaposi’s sarcoma, even though HHV8 is the causative virus. Radiation exposure from radiotherapy has been strongly associated with secondary sarcoma development in certain cancer patients. In fact, the risk of malignant bone tumors increases as the cumulative dose of radiation to the bone increases (p for trend <0.001). A recent meta-analysis reported that children with a history of hernias have a greater risk of developing Ewing’s sarcoma (adjusted OR 3.2, 95% CI 1.9, 5.7). Bone development during pubertal growth spurts has been associated with osteosarcoma development. Occupational factors such as job type, industry, and exposures to chemicals such as herbicides and chlorophenols have been suggested as risk factors for sarcomas. A case-control study found a significant increase in soft tissue sarcoma risk among gardeners (adjusted OR 4.1, 95% CI 1.00, 14.00), but not among those strictly involved in farming. A European-based study reported an increased risk in bone tumors among blacksmiths, toolmakers, or machine-tool operators (adjusted OR 2.14, 95% CI 1.08, 4.26). Maternal and paternal characteristics such as occupation, age, smoking status, and health conditions experienced during pregnancy also have been suggested as sarcoma risk factors and would be important to assess in future studies. The limited studies we identified demonstrate significant relationships with sarcoma risk, but many of these results now require further validation on larger populations. Furthermore, little is known about the biologic mechanisms behind each epidemiologic association assessed in the literature. Future molecular epidemiology studies may increase our understanding of the genetic versus environmental contributions to tumorigenesis in this often deadly cancer in children and adults.

## Introduction

Sarcomas, tumors of putative mesenchymal origin, account for nearly 21% of all pediatric solid malignant cancers and less than 1% of all adult solid malignant cancers [1]. In addition, sarcomas represent multiple malignancies rather than a single cancer [2]. For example, more than 50 distinct histologic sarcoma subtypes exist. Furthermore, many of these subtypes can occur at any age and are not restricted to a specific location of the body. The rarity of the disease combined with the diverse number of subtypes can make sarcomas very difficult to study. In order for the evaluation of the epidemiology and etiology of sarcomas to be feasible, this review will take a broad perspective, noting differences primarily between the two most common and distinct sarcoma groupings, malignant bone tumors and soft tissue sarcomas [2].

Soft tissue sarcomas often form in the body’s muscles, joints, fat, nerves, deep skin tissues, and blood vessels. As the name implies, malignant bone tumors such as osteosarcomas and Ewing’s sarcomas are found throughout the bones of the body, but also can commonly be found in the cartilage [3]. In 2010, the National Center for Health Statistics (NCHS) projected that 10,520 and 2,650 Americans, including all ages, will have been diagnosed with soft tissue and malignant bone tumors, respectively [4]. Furthermore, it is also projected that 3,920 and 1,460 Americans will die in 2010 from soft tissue and malignant bone tumors, respectively.

Sarcomas, although relatively rare, are quite deadly, especially soft tissue sarcomas. The primary reason for this is due to delayed diagnosis and advanced disease, or metastasis, at presentation [3]. Early stage sarcomas lack distinct symptoms that would potentially allow for early diagnosis. In addition to being a deadly disease, sarcomas also occur more frequently in young adults and adolescents compared to other cancers. Thus, despite lower incidence rates, the years of life lost can often be substantial. These facts present adequate evidence that strategies to prevent sarcoma occurrence would prove to be beneficial. However, little scientific knowledge and consensus pertaining to the cause of sarcomas exists. It is evident that further epidemiological research is warranted in order to more clearly define environmental risk factors. The purpose of this review is to perform a thorough evaluation of the existing literature on the epidemiology and etiology of sarcomas. Thus, conclusions will be made on the risk factors that are established, which will be of benefit on drawing conclusions on appropriate preventative guidelines.

## Methods

In order to identify the potential environmental risk factors for sarcomas, we reviewed all published articles that pertained to the epidemiology of sarcomas. In addition, background information on the descriptive epidemiology and basic genetics of sarcomas was also obtained. For the environmental risk factor assessment, we performed a literature search using the PubMed database. Search terms included key words and phrases, such as “epidemiology,” “risk factors,” and “case–control.” In addition, common sarcoma subtype names of both malignant bone tumors and soft tissue sarcomas were also used in combination with our search terms in order to yield the most relevant articles. The PubMed “all related articles” feature was also used in our search for published papers that were related to our topic of interest, which may have not been listed, based on our search terms.

Due to the sparsity of literature on sarcomas, a strict inclusion criterion was not vigorously followed. However, the selected papers used must have followed an epidemiologic study design. Thus, case reports were excluded. All published papers used in this review were also required to have been published after 1980, in order to minimize inaccurate diagnoses conflicts. A total of 29 adult studies and 22 pediatric studies were gathered, reviewed, and included in the environmental risk review (Tables 1 and 2). The majority of the conducted research were case–control studies, but some cohort, ecologic, and case-series studies (>250 cases) were also found and included.

**Table 1:** Adult sarcoma study descriptions

**Location & time**	**Author**	***N*Cases**	***N*Controls/Cohort**	**Risk cactors examined**
**Cohort Studies** UK & France 1942–1986 [5,6]	Le vu et al. (1998), Menu-Branthomme et al. (2004)	OS 32, STS 25	4,400, 4,400	Radiotherapy
Finland 1953–2000 [7]	Virtanen et al. (2006)	STS & MBT 147	295,712	Radiotherapy
France 1954–1983 [8]	Rubino et al. (2005)	STS & MBT 14	6,597	Radiotherapy
Japan 1958–2001 [9]	Samartzis et al. (2011)	MBT 19	120,321	Ionizing radiation- atomic bomb
USA 1973–1995 [10]	Hwang et al. (2003)	STS 135	194,798	Radiotherapy
Amsterdam 1984–1996 [11]	Renwick et al. (1998)	KS 99	3,443	HHV8 infection
**Nested Case–control** UK 1940–1983 [12]	Hawkins et al. (1996)	MBT 59	220	Radiotherapy
International-multiple locations ?-1991 [13]	Kogevinas et al. (1995)	STS 11	55	Phenoxy herbicides, chlorophenols, dioxins
**Case–control** England & Wales 1968–1976 [14]	Balarajan et al. (1984)	STS 1,961	1,961	Agriculture and forestry occupations
Sweden 1975–1982 [15]	Wingren et al. (1990)	STS 96	650	Job type, chemical agents, and other occupational factors
Kansas, USA 1976–1982 [16,17]	Hoar et al. (1986), Zahm et al. (1989)	STS 228, STS 133	1610, 948	Agricultural herbicide use, tobacco use, medical history, occupation
Umea, Sweden 1978–1983 [18]	Hardell et al. (1988)	STS 54	311	Phenoxyacetic acids, chlorophenols
Uppsala, Sweden 1978–1986 [19]	Eriksson et al. (1990)	STS 237	237	Occupation, occupational exposures, dioxins
Wisconsin, USA 1979–1989 [20]	Moss et al. (1995)	OS 167	989	Fluoridated drinking water
Wisconsin, USA 1980–1997 [21]	Guse et al. (2002)	OS 319	3,198	Radium in drinking water
Washington, USA 1981–1984 [22]	Woods et al. (1987)	STS 128	694	Phenoxy herbicides, chlorophenols, and other occupational exposures
Victoria, Australia 1982–1988 [23]	Smith et al. (1992)	STS 30	60	Phenoxy herbicides and chlorophenols
New York & Washington DC, USA 1982-? [24]	Engels et al. (2003)	KS 29	57	Immunologic and virologic factors
Northern Italy 1983–1998 [25]	Fioretti et al. (2000)	STS 104	505	Menstrual and reproductive factors
USA-multiple locations 1984–1988 [26,27]	Hoppin et al. (1998), Hoppin et al. (1999)	STS 295, STS 200 & MBT 51	1908, 1908	Chlorophenols and other occupational exposures
USA-multiple locations 1984-? [28]	Moore et al. (1996)	KS 21	42	Kaposi's sarcoma-associated herpesvirus infection
Northeast Italy 1985–1991 [29-31]	Franceschi et al. (1992), Serraino et al. (1992), Serraino et al. (1991)	STS 93, STS 93, STS 88	721, 721, 610	Occupational factors, tobacco, alcohol, drugs, pesticides, and history of infection
Manua, Italy 1989–1998 [32]	Comba et al. (2003)	STS 37	171	Residence near industrial waste incinerators
Uganda 1994–1998 [33]	Ziegler et al. (2003)	KS 117	1,282	HHV8 infection
Europe-multiple locations 1995–1997 [34]	Merletti et al. (2006)	MBT 96	2,632	Job type and occupational exposures
Sicily, Naples, and Rome 1998–2001 [35]	Goedert et al. (2002)	KS 141	192	Birth order, sexual history, medical history, and cigarette consumption
**Ecologic** France 1980–1995 [36]	Viel et al. (2000)	STS 110	N/A	Residence near industrial waste incinerators
**Case-series** USA-multiple locations 1960–1992 [37]	Cope et al. (2000)	ES 306	N/A	Hernias
Bologna, Italy 1981–2001 [38]	Longhi et al. (2005)	OS 962	N/A	Height, stature, and growth rate

STS = Soft tissue sarcoma, MBT = Malignant bone tumor, KS = Kaposi's sarcoma, OS = Osteosarcoma, ES = Ewing's sarcoma, CS = Chondrosarcoma.

**Table 2:** Pediatric sarcoma study descriptions

**Location & time**	**Author**	**N Cases**	**N Controls/Cohort**	**Risk factors examined**
**Pooled** USA, UK, Sweden, Spain, Italy, Germany^a^ 1945–2001 [39]	Mirabello et al. (2011)	OS 1501	1,501,000^	Height at diagnosis, birth weight
Austrailia and California^b^ 1978–1996 [40]	Valery et al. (2005)	ES 138	574	Hernias
USA-multiple locations (CA, MN, NY, TX, WA) 1980–2004 [41-44]	Spector et al. (2009), Von Behren et al. (2010), Ognjanovic et al. (2009), Johnson et al (2009)	MBT 573 & STS 1067, MBT 550 & STS 1054, RS 583, MBT 511 & STS 1000	57966, 57966, 57966, 57966	Birth weight, birth order, parental age, gestational age, and other birth characateristics
**Meta-analysis** USA & Canada^c^ 1980–2002 [40]	Valery et al. (2005)	ES 357	745	Hernias
**Cohort** Connecticut, USA 1936–1979 [45]	Tucker et al. (1987)	MBT 64	9,170	Radiotherapy (treatment of primary childhood cancers)
**Case–control** Ontario, Canada 1964–1988 [46]	Finkelstein et al. (1996)	MBT 238	432	Radium in drinking water
North Carolina, United States 1967–1976 [47]	Grufferman et al. (1982)	RS 33	99	Parental smoking habits, maternal age, maternal antibiotic use, and vaccination history
Northern England 1968–2000 [48]	Pearce et al. (2007)	MBT 245 & STS 320	29,520	Paternal occupational exposure to electro-magnetic fields
USA-multiple locations 1972-? [47]	Winn et al. (1992)	ES 208	395	Parental smoking habits, hernias, and parental occupational factors
USA 1972–1997 [49]	Grufferman et al. (1993)	RS 322	322	Parental cocaine and marijuana use
Los Angeles, USA 1972–1981 [50]	Operskalski et al. (1987)	OS 64	124	Birth length, gestational age and height at diagnosis
New York State, USA 1978–1988 [51]	Gelberg et al. (1997)	OS 130	130	Birth weight, birth height, and pubertal growth factors
United Kingdom 1980–1983 [52,53]	Hartley et al. (1988), Hartley et al. (1988)	MBT 30 & STS 43, MBT 30 & STS 43	146, 146	Birth weight, pregnancy conditions, antibiotic use after birth
Ontario, Canada 1980–1988 [54]	Hum et al. (1998)	MBT 152	713	Parental occupations
USA & Canada 1983–1987 [55]	Buckley et al. (1998)	OS 152 & ES 153	305	Birth weight, birth height, and pubertal growth factors
United Kingdom 1991–1996 [56]	Smith et al. (2009)	MBT & STS 251	6,337	Birth weight and gender
Austrailia 1991–1996 [57,58]	Valery et al. (2003), Valery et al. (2002)	ES 106, ES 106	344, 344	Parental occupation, hernias, and pubertal growth factors
USA-multIple locations 1992–1995 [59]	Bassin et al. (2006)	139	280	Fluoride levels in drinking water
Germany 1992–1997 [60,61]	Schuz et al. (2007), Shuz et al. (1999)	MBT 97 & STS 137, MBT 97 & STS 137	2057, 2588	Birth weight, family size, maternal age, gestational age, paternal smoking, birth weight for gestational age, and other birth characateristics
USA-multiple locations 1994–2000 [62]	Troisi et al. (2006)	OS 158	141	Birth weight, birth length, birth order, height and weight at diagnosis, and other pubertal growth factors
**Case-series** USA-multiple locations 1972–1978 [63]	Pendergrass et al. (1984)	ES 291	N/A	Adolescence stature: height and weight
United Kingdom 1978–1997 [64]	Cotterill et al. (2004)	MBT 720	N/A	Adolescent height, stature, and growth factors

STS = Soft tissue sarcoma, MBT = Malignant bone tumor, KS = Kaposi's sarcoma, OS = Osteosarcoma, ES = Ewing's sarcoma, CS = Chondrosarcoma, RS = Rhabdomyosarcoma.

^2000 U.S. National Center for Health Statistics Simulated Controls.

Already referenced studies included in pooled/meta analyses: ^a^[41,62,64]^b^[57]^c^[54,65].

## Descriptive epidemiology

**Figure 1:**
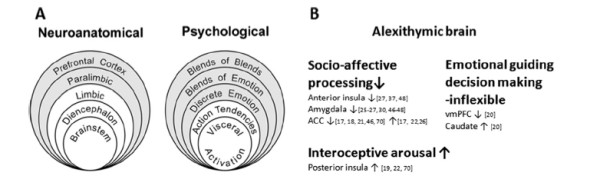
**Distribution of new sarcoma cases by histology (2008)**.

In SEER data (1973–2008), we observed that soft tissue sarcomas currently occur much more frequently than malignant bone tumors [1]. In 2008, soft tissue sarcomas accounted for nearly 87% of all sarcomas diagnosed, while the remaining 13% of the diagnoses were malignant bone tumors [1]. Osteosarcomas and chondrosarcomas were the most commonly diagnosed malignant bone tumors, accounting for over half of all the malignant bone tumor diagnoses. According to SEER, “other specified soft tissue sarcomas” accounted for roughly 51% of all sarcomas diagnosed in 2008, and clearly lead soft tissue sarcoma occurrence. Fibrosarcomas and Kaposi sarcomas were the two distinct and individual soft tissue subtypes identified, and predominantly diagnosed in 2008, accounting for roughly 7% and 9% of all sarcoma diagnoses respectively (Figure 1) [1]. 

**Figure 2:**
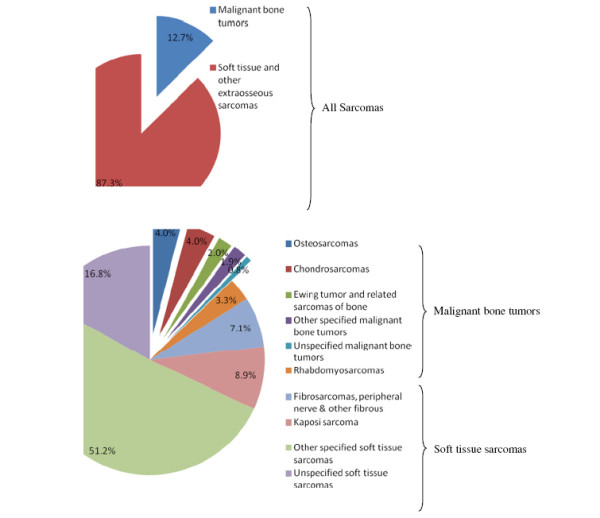
**Distribution of ages at diagnosis, 2004-2008**.

**Figure 3:**
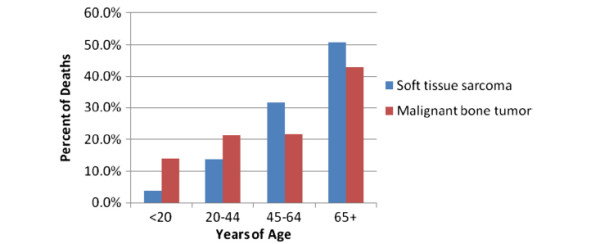
**Distribution of ages at death, 2003-2007**.

**Figure 4:**
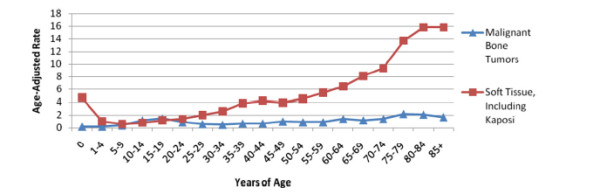
**Incidence of malignant bone tumors vs. soft tissue sarcomas by age (2004-2008)**.

Age is an important determinant of sarcoma occurrence. Based on current statistics provided by the NCHS and SEER, from 2004–2008, the mean age at diagnosis for soft tissue sarcomas and malignant bone tumors was 58 and 40 years of age, respectively [4]. From 2003–2007, the mean age at death for soft tissue sarcomas and malignant bone tumors was 65 and 58 years of age, respectively. For further details on the distribution of ages at time of diagnosis and death, please refer to Figures 2 and 3. Generally, an increase in the rate of soft tissue sarcomas occurs in new born babies and young children, until they reach the age of 5 [1]. Young adults experience the lowest incidence of soft tissue sarcomas, but occurrence steadily increases until the age of 50. At ages greater 50 years and above, incidence of soft tissue sarcomas increases much more dramatically. Malignant bone tumors, generally have a fairly stable rate of incidence across all ages. However, noticeable increase in rates often occur in adolescents and young adults due to osteosarcoma and Ewing’s sarcoma. Moderate increases in bone tumor incidence also tend to occur in people in their 70s and 80s (Figure 4) [1]. 

## Race & geography

Ewing’s sarcoma is a relatively rare bone tumor with limited epidemiologic data; however one of the few well-described risk factors for this specific type of cancer is race. It is known that whites are predominantly affected by Ewing’s sarcomas, whereas incidence rates in Asian and African populations are often considerably less [66]. This difference in incidence by race, suggests a genetic component to Ewing’s sarcoma, which has led researchers to believe that that these racial differences are biologically true. Jawad et al. (2009), found a 9-fold significant difference in Ewing’s sarcoma rates between Caucasians and African Americans, clearly portraying that Caucasians are at greater risk for this particular sarcoma [67]. Racial disparities exist among other sarcoma subtypes as well. For example, rates of chondrosarcomas are often found to be higher among American populations, versus, people living in Asian countries [68].

We further attempted to investigate these racial relationships by assessing sarcoma incidence across several geographical regions. *Cancer Incidence in Five Continents*, *Volume IX,* published by the International Agency for Research on Cancer (IARC), provides an in-depth look at sarcoma incidence by continent, country, and small region [69]. Age-Standardized osteosarcoma incidence rates for both males and females did not appear to drastically differ between Asian countries and the United States as some investigators have reported [68]. Overall, it appears that sarcoma incidence rates are comparable throughout much of the world. However, there are some instances where notable differences exist. Japanese males living in the state of California, have a reported osteosarcoma incidence rate of 1.3 per 100,000 males [69]. This rate is relatively high in comparison to incidence rates observed through much of the world. Typically, osteosarcoma incidence rates range from 0.2-0.6 per 100,000 males, depending on geographic region. Osteosarcoma incidence rates of similar magnitude were not observed throughout Japan. However, a high incidence of 1.1 cases per 100,000 was reported among Japanese males living in Hawaii [69]. These findings may suggest that Japanese migrants living in “westernized” regions may be subject to increase osteosarcoma risk due to environmental or lifestyle factors. A relatively high osteosarcoma incidence rate of 1.4 per 100,000 females was also reported in Sondrio, Italy. The remaining geographic regions of Italy reported significantly lower osteosarcoma incidence rates among females, ranging from 0.1-0.4 per 100,000. These geographical differences in sarcoma incidence clearly warrant the need for further investigation, which goes beyond the scope of this review. These findings reflect that both genetic and environmental factors likely contribute to the etiology of sarcomas.

The literature available on racial differences in regards to soft tissue sarcomas appeared to be lacking and required us to further analyze the SEER database for racial disparities. We generated soft tissue sarcoma incidence rates, which included years 1973–2008, and found that Blacks had the highest overall incidence rate of 5.1 per 100,000 (the opposite of Ewing’s sarcoma, a bone tumor sarcoma) [1]. Whites’ had an incidence rate of 4.5 per 100,000, followed by American Indian/Asian Pacific Islanders, with a rate of 2.8 per 100,000. This is evidence to show that race also influences disease occurrence among those with soft tissue sarcomas. Further investigation of the biologic and genetic differences in sarcoma tumors by race is needed in order to gain greater understanding of the potential mechanisms responsible for these mentioned racial differences.

## Genetics

Several different inherited genetic syndromes increase the risk for subsequent sarcoma development. Some of the most well-known syndromes are neurofibromatosis (NF1), also known as von Recklinghausen’s disease, Li-Fraumeni syndrome (LFS), and Retinoblastoma (Rb) [2]. Individuals diagnosed with NF1 have a 10% cumulative lifetime risk of developing malignant peripheral nerve sheath tumors (MPNST) [70]. NF1 results from an autosomal dominant process that leads to improper function of the *NF1* gene, which is responsible for producing Neurofibromin. Neurofibromin ultimately functions as a tumor suppressor gene through guanosine triphosphatase (GTPase) activity from the proto-oncogene, Ras. Thus, loss of function of the *NF1* gene, leads to increased Ras activity, promoting tumor development.

Li-Fraumeni syndrome (LFS) was one of the first cancer genetic syndromes discovered to have a strong association with sarcomas [25]. In fact, LFS was initially clinically defined as having “a proband who had a sarcoma diagnosed before 45 years of age and a first-degree relative who had any cancer under 45 years of age and a first- or second-degree relative who had any cancer under 45 years of age or a sarcoma at any age [47]." Newer definitions for LFS are based on the “Chompret Criteria” which define the diagnosis as: “Proband with LFS tumor (eg., soft tissue sarcoma, osteosarcoma, brain tumor, premenopausal breast cancer, adrenocortical carcinoma, leukemia, lung brochoalveolar cancer) before age 46 years AND at least one first- or second-degree relation with LFS tumor before age 56 years or with multiple tumors; OR proband with multiple tumors (except breast), two of which belong to LFS tumor spectrum and first of which occurred before age 46 years; OR patient with adrenocortical carcinoma or choroid plexus tumor” [65]. The syndrome results from germline (constitutional) mutations in the tumor suppressor gene, *TP53*[52]*.* The *TP53* tumor suppressor gene is responsible for inhibiting cell growth and stimulating cell apoptosis, as well as DNA repair [2]. Thus, mutations in *TP53* can lead to the early development of sarcomas and other tumors through the acquisition of genomic instability. In fact, children with the soft tissue sarcoma rhabdomyosarcoma presenting at less than 3 years of age appear to have an increased likelihood of harboring *TP53* germline mutations [39,56]. It is also important to note that roughly 30-60% of non-LFS soft tissue sarcomas will have somatic mutations of the *TP53* gene [53].

Retinoblastoma (Rb), hereditary or non-hereditary, is a relatively rare tumor that develops in the retinal cell found in the eye. Hereditary Rb survivors have a greater risk of developing secondary malignancies, in particular osteosarcoma [60]. It was recognized early on that radiation treatment further increased the risk for secondary malignancies among Rb survivors. When possible, radiation is now avoided in Rb treatment strategies. However, patients with hereditary Rb are still at increased risk for other tumors even without radiation exposure (including bone and soft tissue sarcomas, brain tumors, nasal cavity cancer, melanoma, lung, gastrointestinal, and bladder cancer) and this risk increases as Rb patients continue to age [41,50]. Rb develops by means of germline mutations that lead to inactivation of an allele in the tumor suppressor gene, *RB1*. It has been estimated that Rb survivors have a 500-fold increase incidence of osteosarcomas as compared to the general population [42].

Osteosarcomas, in particular, seem to be associated with hereditary cancer syndromes [43]. Other familial predisposition syndromes associated with osteosarcoma risk include the very rare, autosomal recessive DNA helicase syndromes including: Rothmund Thomson II (*REQ4* mutations), RAPADILINO Syndrome (RA: RAdial aplasia or hypoplasia, PA: PAtellae aplasia or hypoplasia and cleft or high arched PAlate, DI: DIarrhea and DIslocated joints, LI: LIttle size and LImb malformations, NO: long, slender NOse and NOrmal intelligence, *REQ4* mutations), Werner (*WRN* mutations), and Bloom Syndrome (*BLM* mutations). In addition, inherited defects in ribosomal proteins lead to the autosomomal dominant syndrome of Diamond-Blackfan Anemia (*RPS19, RPL5, RPL11, RPL35A, RPS24, RPS17, RPS7, RPS10,* and *RPS26* mutations) which has been associated with rare cases of osteosarcoma.

Ewing’s Sarcoma, on the other hand, is currently not associated with any known gene mutations or hereditary cancer syndromes [44]. Nevertheless, the associations described below with race and familial hernia risk suggests a yet undefined genetic association. A recent publication has reported that common variants near *TARDBP* and *EGR2* are associated with susceptibility to Ewing’s sarcoma [61]. As research continues into the etiology of Ewing’s sarcoma, it can be expected that more genetic risk factors will be identified.

## Etiologic studies

Investigations of the potential risk factors for sarcoma occurrence commonly share similar study characteristics. Even though many different sarcoma subtypes exist, they are commonly grouped together and studied as a single outcome because of the rarity of sarcoma occurrence. However, studies will commonly separate adult and pediatric cases, due to the potential for differences in their etiologic properties. Furthermore, case–control studies are the standard approach for assessing many of the environmental risk factors largely due to the rarity of sarcomas. This study design, commonly used as the method for investigating rare outcomes, can discourage investigators and reviewers from making strong conclusions. Case–control studies are simply prone to more bias than prospective studies and this has been evident while reviewing the literature. The majority of sarcoma-related case–control studies are relatively small and cover a wide-range of exposures and carcinogenic factors. The following report contains a comprehensive review of the major environmental risk factors that have been investigated.

### Female Hormones and Reproductive Factors (Adult Sarcomas)

Very few studies have assessed the potential role of female hormones on sarcoma development. One case–control study in Northern Italy investigated the potential association across a wide array of female hormone related factors [37]. There were 104 soft tissue sarcoma cases and 505 controls available for analysis, but no significant associations were reported based on menstrual cycle patterns, age at menopause, parity, and number of abortions. The only suggestive association was for women who had become pregnant with their first child at later ages (>29 years of age) (adjusted OR = 3.16, 95% CI 0.96, 10.44).

### Prenatal characteristics (Pediatric sarcomas)

Three studies assessed the relationship between specific prenatal characteristics and sarcoma occurrence [40,51,57]. Winn et al. (1992) reported in a case–control study of 208 cases and 395 sibling/regional controls, that women who gave birth to children that later developed Ewing’s sarcoma were more likely to have used medications for nausea and vomiting during their pregnancy (adjusted OR = 2.6, 95% CI 1.2, 5.9) [40]. However, this result was only found to be significant where siblings acted as study controls. Such an association was not seen where regional controls were used. Thus, this result could reflect selective recall bias by the parent for the case child. Grufferman et al. (1982) conducted a case–control study in North Carolina, comprised of 33 cases and 99 controls, and reported that mothers who had used antibiotics during or closely preceding pregnancy (adjusted RR =2.7, 95% CI 1.1, 6.4), or had experienced an overdue or assisted delivery (adjusted RR = 2.6, 95% CI 1.1, 7.1) were at increased risk of giving birth to a child that would later develop the soft tissue sarcoma known as rhabdomyosarcoma [57]. Interestingly enough, a study based in the United Kingdom, comprised of 73 cases (43 soft tissue/30 malignant bone) and 146 controls reportedly found that soft tissue sarcoma occurrence in children has also been associated with antibiotic use in children closely after birth (adjusted RR = 6.81, 95% CI 1.13, 71.18) [51]. This study also examined the pregnancy condition known as toxemia and found that it increased the risk of soft tissue sarcomas (adjusted RR = 2.71, 95% CI 1.05, 7.06). However, it is important to note that criteria for the toxemia case mothers was based on recorded pregnancy symptoms such as hypertension, edema, and albuminuria, rather than an actual record of having been diagnosed with toxemia.

### Birth characteristics (Pediatric sarcomas)

Many studies have assessed the potential relationship between pediatric cancer risk and many different birth factors. A recent study including 251 pediatric sarcoma cases and 6,337 controls investigated birth weight as a risk factor for pediatric sarcomas but no significant association was observed [64]. The previously mentioned case–control study in the United Kingdom with 73 cases and 146 controls reported some evidence suggesting that a potential relationship might exist between Ewing’s sarcoma and birth weight [51]. They reported that Ewing’s sarcoma cases had a median weight of 3,015 g compared to the controls which had a median weight of 3,400 g (p = 0.02). A pooled analysis included 434 osteosarcoma cases and 1,000 controls studies, reported a significant association between osteosarcoma risk and high birth weight (> = 4,046 g), compared to an average birth weight (2,665-4,045 g) (adjusted OR = 1.35, 95% CI 1.01, 1.79) [38]. The biological mechanisms that define the relationship between birth weight and childhood sarcomas are not clear [64]. Birth weight has also been assessed to be possibly associated with the risk of soft tissue sarcomas and other non-osteosarcoma bone tumors. However, no major statistically significant results were observed among these studies [55,62-64].

Gestational age has also been investigated as a potential factor that may be associated with an increased risk of pediatric sarcomas. A case–control study made up of 64 cases and 124 controls reported that osteosarcoma risk increased among those born a week early (OR = 2.8, 95% CI 1.1-6.8) [24]. A published reported based on a large pooled analysis of 573 malignant bone cases and 57,966 controls, also found an increased risk of Ewing’s sarcoma among those cases that had a recorded gestational age of 32–36 weeks, versus those classified as being born after 36 weeks of gestation (adjusted OR =1.68, 95% CI 1.03-2.76) [63]. This pooled analysis linked birth and cancer registry data across five U.S. states, which allowed for the large sample size. Furthermore, a recent case–control study in Germany with 97 malignant bone cases and 137 soft tissue sarcoma cases found no significant increase in sarcoma risk among those classified as small-for-gestational age, nor those classified as being large-for-gestational age [55].

Birth order and maternal age have also been investigated as risk factors for sarcoma. The previously mentioned multi-U.S. pooled analysis with 57,966 controls, included 583 rhabdomyosarcoma cases. A decreased risk in rhabdomyosarcoma was reported, among third born children, where the firstborn child was classified as the comparison group (adjusted OR = 0.70, 95% CI 0.54, 0.91) [28,71]. The same pooled analysis reported that the risk of rhabdomyosarcoma increases among cases with mothers advancing in age (per 1-year increase, adjusted OR =1.03, 95% CI 1.01, 1.04) (per 5-year increase, adjusted OR = 1.19, 95% CI 1.05, 1.34) [11,28]. Another study including 137 soft tissue sarcoma cases and 2,588 controls reported an actual decrease in risk among cases with older mothers (>35 years) (OR = 0.4, 95% CI 0.1, 1.0) [33]. In addition, a significant increase in risk was observed for cases who had young mothers. (<20 years) (OR = 2.2, 95% CI 1.0, 4.7). All soft tissue sarcomas were also grouped together in this analysis which ultimately may not share common etiologic properties, thus leading to invalid conclusions. Studies have also observed increasing linear trends of sarcoma risk, based on incremental increases in paternal age [11,28].

Inguinal and umbilical hernias are a commonly studied birth anomaly that has been found to be associated with Ewing’s sarcoma [14,15,29,40]. The previously mentioned case–control study conducted by Winn et al. (1992) reported that umbilical and inguinal hernias, occurring early in life, are diagnosed six times more frequently in Ewing’s sarcoma cases than controls [40]. A recent meta-analysis of 357 cases and 745 controls, which included the study by Winn et al. (1992) [40] and two additional case–control studies, also reported that children with a history of hernias have a greater risk of developing Ewing’s sarcoma (adjusted OR 3.2, 95% CI 1.9, 5.7) [15]. Cope et al. (2000) reported in a case-series study of 324 cases, a significantly higher relative risk of inguinal hernias among Ewing’s sarcoma cases compared to population estimates (females, RR = 13.3, 95% CI 3.6, 34.1) (males, RR = 6.67, 95% CI 2.67, 13.7) [15]. Authors’ hypothesized that these findings suggest a disruption in normal embryological development, which perhaps may relate to an *in utero* exposure or indicate an underlying genetic disorder.

### Growth and development in early adolescence (Pediatric sarcomas)

Several studies have concluded that having a tall stature or experiencing an earlier pubertal growth spurt may be important factors in the etiology of osteosarcomas [16,22,30,34,38]. Out of the six papers reviewed, three were case–control studies [22,24,30], two were case-series studies [16,34], and one study conducted a pooled analysis [38]. Only one paper did *not* report an association between osteosarcoma and increased growth in early adolescence [24]. This paper was based on a previously mentioned case–control study that had assessed gestational age and osteosarcoma risk. This case–control study compared to the other reviewed studies was relatively small, with only with 64 cases and 124 controls. Both case-series studies had over 700 cases and the case–control studies had at least double the number of osteosarcoma cases to analyze. Thus, the small sample size may be one reason why the results were not similar to the other studies. Individuals with osteosarcoma, are commonly found to be taller than the general population near the time of diagnosis [16,30,34,38]. It can be concluded that rapid bone development during the pubertal time window may lead to an increased risk of osteosarcoma [38]. Further investigation is needed in order to understand the physiologic mechanisms that are responsible for this relationship. Buckley et al. 1998 found that the timing of pubertal development may also be an important factor in osteosarcoma risk, especially among females [18]. Female cases with osteosarcoma tended to experience breast development (11.4 vs. 11.8 years, P = 0.03) and menarche (12.1 vs. 12.5 years. P = 0.002) at significantly earlier times in their adolescent lives, compared to controls. Two studies also reported that females are generally diagnosed with osteosarcomas at younger ages than males [16,34]. Many of these studies also examined the relationship of growth and development factors in early adolescence with the risk of Ewing’s sarcoma. However, neither height nor weight was found to be associated with Ewing’s sarcoma [23,34]. No studies were found in our search that assessed the relationship between growth and development in early adolescence and soft tissue sarcomas.

### Infection (Adult sarcomas)

It has long been known that people living with AIDS are at very high risk for developing a soft tissue sarcoma known as Kaposi’s sarcoma. In fact, people with AIDS have a 100,000 fold greater risk of developing Kaposi’s sarcoma [19]. However, AIDS does not cause Kaposi’s sarcoma nor is it required to be HIV-positive in order to develop Kaposi’s sarcoma [13]. In recent years, studies have attempted to narrow the causal pathway for Kaposi sarcoma, which has successfully led researchers to the identification of the virus primarily responsible for Kaposi’s sarcoma [13,26,27,54]. Evidence has shown that the human herpes virus 8 (HHV8), a sexually transmitted virus, is strongly associated with an increased risk of Kaposi’s sarcoma in both HIV-positive and HIV-negative individuals. HHV8 is the greatest predictor of Kaposi’s sarcoma development, thus leading researchers to believe that HHV8 plays a central role in the causal pathway for developing Kaposi’s sarcoma.

The potential association of sarcoma development and infections other than HHV8 appears to have been rarely studied. However, one study with 93 cases and 721 controls examined the potential risk of other viral infections on soft tissue sarcoma development [58]. They reported a greater risk of soft tissue sarcomas among those individuals who had a history of herpes zoster infection (adjusted OR = 2.3, 95% CI 1.1, 4.9), chicken pox (adjusted OR = 2.1, 95% CI 1.2, 4.1), and mumps (adjusted OR = 2.0, 95% CI 1.1, 3.8). Caution must be taken in drawing strong conclusions from a single paper and a topic that appears to have been rarely studied. Nevertheless, the association between viruses and sarcoma warrants further consideration.

### Job type, industry, and occupational exposures (Adult sarcomas)

Occupational factors such as job type and industry have been among the most frequently studied risk factors in sarcoma research. Balarajan et al. (1984) reported that farmers, farm managers, and market gardeners have a significant increase in risk for developing soft tissue sarcomas. (adjusted OR 1.7, 95% CI 1.00, 2.88) [48]. However, other studies did not find a significant increased risk among those employed in all agriculture-based positions [8,45]. For example, one study of 96 cases and 650 controls found a significant increase in soft tissue sarcoma risk among gardeners (adjusted OR 4.1, 95% CI 1.00, 14.00), but not among those strictly involved in farming [8]. Other occupations such as railroad and construction work were also suggestive of an increase in risk of soft tissue sarcomas, but these relationships have not been shown to be statistically significant. The varying results among studies could potentially be due to the differences in epidemiologic methodology. For example, the results Balarajan et al. (1984) reported were based primarily on registry data, relying upon occupational coding in order to study these relationships, while the other two studies, which did not find similar significant associations, relied upon questionnaires and other similar tools for obtaining occupational information.

The association between malignant bone tumors and occupation has not been studied as often as soft tissue sarcomas. One European based-study, consisting of 96 cases and 2,632 controls, reported an increased risk in bone tumors (includes osteosarcomas and chondrosarcomas) among those who worked as blacksmiths, toolmakers, or machine-tool operators (adjusted OR 2.14, 95% CI 1.08, 4.26) [12]. This study also found that individuals involved in bricklaying (adjusted OR 2.93, 95% CI 1.55, 5.53) and carpentry, (adjusted OR 4.25, 95% CI 1.71, 10.50) were found to be at increased risk for bone tumor development. In addition, this study also reported that cases involved in the manufacturing of wood, cork products, and straw were found to have a significantly increased risk of malignant bone tumor development (adjusted OR 3.58, 95% CI 1.70, 7.56). Individuals classified as manufacturers of machine and equipment, were also found to be at greater risk for bone tumors (adjusted OR 2.02, 95% CI 1.00, 4.08). Interestingly, the study participants classified as being in the industry of agriculture, growing of crops, and other related fields, which might be frequent users of herbicides and pesticides, were not found to be related to bone tumor development [12].

The Nordic Occupational Cancer (NOCCA) project has collected roughly 45 years of cancer incidence data by occupational category for Denmark, Finland, Iceland, Norway, and Sweden [5]. Standardized incidence ratios (SIR) were utilized in describing the relationship between cancer incidence and occupation. An SIR is used to determine whether the number of observed cases of cancer is higher or lower than expected, given the age distribution and population under study. This NOCCA reported a statistically significant elevated SIR of malignant bone tumors among men classified as “other health workers.” (SIR 2.25, 95% CI 1.29, 3.66). This may suggest that health-related occupations such as radiologists and health technologists may be at increased risk for bone tumor development due to radiation exposure. Statistically significant elevated SIRs were also reported among men classified as military workers (SIR 2.88, 95% CI 1.68, 4.61), seamen (SIR 1.92, 95% CI 1.05, 3.22), and drivers (SIR 1.45, 95% CI 1.09-1.88). Further investigation will be needed in order to determine if these particular occupations are associated with ionizing radiation and other physical elements that have been shown to increase the risk of malignant bone tumors. Elevated soft tissue sarcoma SIRs were seen in men, classified as building caretakers (SIR 1.30, 95% CI 1.08, 1.56) and military personnel (SIR 1.27, 95% CI 1.01, 1.59). There were no reported statistical significant SIRs for women.

Besides job type and industry, several studies have assessed the actual exposure to specific chemicals and pesticides at the occupation by administering detailed questionnaires or through personal interviews. Six case–control studies assessed the relationship between herbicide and chlorophenol exposure with soft tissue sarcoma risk, but did not find significant relationships [6,7,9,10,45]. On the other hand, two case–control studies conducted in Sweden and in the U.S., and one international nested case–control study, found a strong relationship between these chemical exposures and soft tissue sarcoma [20,46,59]. We believe these conflicting findings are due to the methodological limitations such as low statistical power, small sample sizes, proxy interviews, and the potential for multiple comparison issues because of the many occupational categories analyzed. Hoppin et al. (1998) conducted the U.S. based case–control study, which included 295 sarcoma cases and 1,908 controls that overcame many of these mentioned weaknesses [46]. They reported a statistically significant relationship between soft tissue sarcoma risk and ever having high-intensity chlorophenol exposure (adjusted OR = 1.79, 95% CI 1.10, 2.88). In fact, among highly exposed subjects, risk increased as the duration of the chlorphenol exposure increased (*p* for trend <0.001). Furthermore, complete occupational histories, spanning multiple years, were obtained from the actual study participants rather than their proxies, which improved the studies ability to successfully analyze duration-response relationships with greater accuracy. In addition, Hoppin et al. (1999) also reported that exposure to cutting oils increased the risk of soft tissue sarcomas, but not bone tumors. (adjusted OR 1.65, 95% CI 1.04, 2.61) [21].

Malignant bone tumors and occupational exposures have not been studied as heavily as the soft tissue sarcomas, although among the limited papers published, no significant relationships were identified [12,21]. Other occupational exposures such as solvents, wood dust, asbestos, DDT, and benzene have also been studied, but none were found to be significantly associated with sarcoma risk [21,45,59].

### Parental occupation (Pediatric sarcomas)

Only a few studies have examined the potential risks of parental occupation on the development of cancer in the offspring. A study in Ontario, Canada, of 152 cases and 713 controls, reported that the risk of Ewing’s sarcoma was significantly elevated among children whose fathers worked in the social sciences (adjusted OR = 6.2, 95% CI 1.6, 24.5) [32]. In addition, the same study reported a greater risk of Ewing’s sarcoma in mothers who were teachers (adjusted OR = 3.1, 95% CI 1.1, 8.7). Investigators found it difficult to hypothesize the possible mechanisms behind these relationships, because these particular occupational settings do not appear to expose the parents to hazardous substances. According to authors, it may be possible that these findings are being influenced by socioeconomic status. However, this study was not able to adjust on this potential confounding factor because data was not available on parental education and income. Three other case–control studies also have reported that a potential relationship exists between farming and the development of Ewing’s sarcoma in offspring [32,36,40]. The case–control study in Australia of 106 cases and 344 controls reported that fathers who worked on a farm at conception or time of pregnancy had offspring with a 3.5 fold greater risk of developing Ewing’s sarcoma, which was statistically significant [36]. This conclusion was only drawn based on offspring that were diagnosed before the age of 20. These conclusions support the general consensus that many of the pesticides and chemicals used in farming are carcinogens and lead to sarcoma development.

The previously mentioned case–control study in Ontario, Canada also investigated the osteosarcoma bone tumor risk in offspring with fathers who were farmers, mothers involved in managerial and administrative work, and mothers involved in product fabricating, assembling, and manufacturing, but did not report any associations [32]. A very large case–control study in Northern England of 565 sarcoma cases and 29,520 controls, reported that parental occupations that involved exposure to electromagnetic fields and non-ionizing radiation were associated with increased risk in chondrosarcomas, a malignant bone tumor subtype (adjusted OR = 8.7, 95% CI 1.55, 49.4) [17]. This particular bone tumor subtype is relatively rare and has not been extensively studied. Caution must be taken in drawing conclusions based on this single study, until similar results are replicated elsewhere. In addition, the potential mechanism(s) responsible for such an association is unclear, but researchers believe low doses of non-ionizing radiation may result in pre-conceptional carcinogenic effects.

### Radiation (Adult & pediatric sarcomas)

High doses of radiation are known to strongly increase the risk of *both* soft tissue sarcomas and malignant bone tumors [31]. This association is primarily reflected by the increase in the number of secondary sarcoma cancers diagnosed among individuals that have been treated by radiotherapy. Several studies have examined this pattern and relationship between primary cancers and secondary development of sarcomas and have found ionizing radiation exposure from radiotherapy to be the key influential factor [31,35,49,72-75]. Virtanen et al. (2006) reported that radiotherapy appears to be associated with an increased risk of developing sarcomas, especially among younger patients under the age of 55 (SIR =4.2, 95% CI 2.9, 5.8) [75]. Furthermore, Hawkins et al. (1996) reported that the risk of malignant bone tumors also increased as the cumulative dose of radiation to the bone increased (*p* for trend <0.001) [49]. Le Vu et al. (1998) implemented a case–control study within a childhood cancer cohort of 4,400 3-year survivors of a first solid cancer and also found that the risk of a secondary bone tumor (osteosarcoma) to be a linear function of the local dose of radiation received [72].

Studies on other potential sources of low dose ionizing radiation and sarcoma risk are relatively infrequent. However, one recently published study that followed atomic bomb survivors from 1958 to 2001 reported that lower doses of ionizing radiation increased the occurrence of bone sarcoma diagnoses (RR = 7.5 per Gy, 95% CI 1.34, 23.14) [76]. However, this conclusion was based only on the development of 19 cases during the cohort study period.

### Drinking water (Adult & pediatric sarcomas)

Fluoride exposure in drinking water has been studied as a potential risk factor in the development of osteosarcomas. Fluoride is known to act as a mitogen, increasing the proliferation of osteoblasts and the uptake of fluoride in the bone during periods of growth [77]. This leads to the plausible theory that fluoridated water exposure to individuals during times of growth could be associated with osteosarcomas. The topic has not been extensively studied and conflicting results exist. Moss et al. (1995) reported no significant association in a study of 167 cases and 989 controls [78]. Bassin et al. (2006) reported from a study of 139 cases and 280 controls that a greater risk of osteosarcoma occurrence was seen only in males [77]. Bassin et al. (2006) limited their analysis to include only those cases under 20 years of age, while Moss et al. (1995) included cases of all ages, which likely explains the conflicting results.

Radium at relatively high doses is known to cause malignant bone tumors, but risk assessment of radium at lower doses, appears also to be conflicting and infrequently studied [79]. An ecologic study conducted in Wisconsin, which classified radium exposure in drinking water by average levels observed in each zip code, found no association between osteosarcoma risk and corresponding zip codes that reportedly had higher radium concentrations in the water compared to other surrounding areas [80]. A population-based case–control study, which included 238 cases and 438 controls, also explored the relationship between radium in drinking water and bone tumor risk. They reported a moderate increased risk of osteosarcomas in individuals that had higher radium levels in their water at birth place. (adjusted OR = 1.77, 95% CI 1.03, 3.00) [79].

### Other environmental risk (Adult sarcomas)

Industrial waste incinerators are known to release high emissions of dioxins. Dioxins have been classified as carcinogens, but little is known about the potential risks lower doses of dioxin exposure may have on a population [81]. Two studies examined the relationship between low dose dioxin exposure from incinerators and soft tissue sarcoma risk. Comba et al. (2003) reported an elevated risk of soft tissue sarcomas for those whose residence was within 2 km of the incinerator (OR 31.4, 95% CI 5.6, 176.1) [81]. However, caution must be taken with the interpretation of this result since it was based on only 5 exposed cases. Viel et. al (2003) reported the identification of a significant cluster of soft tissue sarcoma cases, that were closest in proximity to the incinerator out of all the geographic regions included in the analysis [82]. This conclusion was based on spatial scan statistic techniques, which successfully identified an excess of 14 observed cases that lived near the incinerator plant.

### Tobacco, alcohol, and drug use (Adult & pediatric sarcomas)

A few studies assessed the risks tobacco, alcohol, and other drugs may have on the development of sarcomas. A case–control study in Kansas, based on 228 cases and 1,610 controls, found a greater risk of soft tissue sarcomas among those that chewed tobacco (adjusted OR = 1.8, 95% CI 1.1, 2.9) [83]. However, an additional study of 93 cases and 721 controls, which also examined the relationship between soft tissue sarcomas and tobacco and alcohol, did not find a significant association between these factors [84]. In addition, a case–control study consisting of 141 cases and 192 controls, found that Kaposi’s sarcoma risk *decreased* among those that smoked cigarettes [85]. In fact, a dose–response relationship was observed, where as the consumption of cigarettes smoked per day increased, the risk of developing Kaposi’s sarcoma continually decreased (*p* for trend <0.001). Further study result replication would be needed in drawing strong conclusions on this potential inverse relationship. However, the investigators did mention that this identified relationship could have been influenced through participation bias. An unbiased control sample was pursued, but it was reported that the prevalence of male smokers enrolled as controls was higher than expected for the study area (86%).

We also retrieved a few studies on the potential risks that parental smoking and recreational drug use might confer on sarcoma development in children. One study found that the risk of Ewing’s sarcoma rose with the number of cigarettes the mother smoked during pregnancy [40]. However, this association was only seen when siblings were used as controls, rather than regional controls, which as previously mentioned, could reflect selective recall bias by the parent for the case child. An additional paper, which assessed both maternal and paternal smoking habits did not find any significant associated risks [33]. One particular case–control study, of 322 cases and 322 controls, found that parents’ use of marijuana and cocaine during the year prior to their child’s birth may increase the risk of developing rhabdomyosarcoma by 2 to 5 fold [86]. Similar to the other findings described thus far, further studies must be implemented to draw a consensus on such results.

## Discussion

The studies included in this review have analyzed various potential risk factors for sarcoma development (Table 3). The majority of the assessed exposures lacked enough evidence needed to draw strong conclusions, because these exposures have not been adequately studied. More frequently studied exposures, which were found to be significantly associated with sarcoma occurrence in the majority of circumstances, suffered from the occasional inconsistent result. In these circumstances, we could conclude that suggestive evidence of an association existed. If an exposure had been studied extensively and the results from these studies were overwhelmingly consistent then we classified these exposures as being strongly associated with sarcomas. HIV-positive individuals are clearly at an increased risk for Kaposi’s sarcoma, even though it has been recently discovered that HHV8 is the particular virus known to be central in the causal pathway. Furthermore, radiation exposure by means of radiotherapy has been shown to be strongly associated with secondary sarcoma development. This evidence has been replicated several times in several different studies as previously discussed and has been demonstrated to be quite consistent [31,35,49,72-75]. The risk of radiation exposure and sarcoma also explains the increase in risk for secondary cancers among those who had been diagnosed with childhood cancer. Such evidence suggests precaution must be taken with radiation exposure during cancer treatment and effective early cancer surveillance strategies must be implemented for early detection of radiation-induced secondary malignancies. Other effective treatment options must continually be investigated to further reduce the risk of sarcomas as secondary tumors. 

**Table 3:** Summary of findings

**Strong evidence of association**	**Cancer**	**Suggestive evidence of association**	**Cancer**	**No evidence of association**	**Cancer**	**Too little evidence to draw conclusions**	**Cancer**
HIV/HHV8 Infections (A) [14,15,29,30,33,34]	KS	Hernias (P) [38,53,62,64]	ES	Menstrual and reproductive factors (A) [56]	STS	Birth weight (P) [41,50,60]	STS, MBT
Radiotherapy (secondary sarcomas) (AP) [6,7,9,20,21,32,46,59]	STS, MBT	Adolescence growth & pubertal factors (P) [24,28,50,55,63,71]	OS	DDT, asbestos, wood dust (A) [18,45,58]	STS, MBT	Birth order (P) [37,57]	RS
		Occupation: job type/industry (A) [16,19,23]	STS, MBT	Flouride in drinking water (AP) [17,36]	OS	Maternal age (P) [37,40,51,57]	STS, RS
		Herbicides & chlorophenols (A) [8,48,58]	STS			Pregnancy medications (P) [39,53,60]	STS, ES, RS
		Place of residence, industrial emissions (A) [49,72]	STS			Pregnancy conditions (P) [39,53,60]	STS
						History of infection: chicken pox & mumps (A) [34]	STS
						Parental occupation (P) [5,10,12,53]	MBT
						Radium in drinking water (AP) [31,35]	OS
						Ionizing radiation -low dose (A) [32]	MBT
						Tobacco, alcohol, & drug use (AP) [51,53,73-76]	STS, RS, KS, ES

STS = Soft tissue sarcoma, MBT = Malignant bone tumor, KS = Kaposi's sarcoma, OS = Osteosarcoma, ES = Ewing's sarcoma, CS = Chondrosarcoma, RS = Rhabdomyosarcoma.

(A) = Adult sarcomas, (P) = Pediatric sarcomas, (AP) = Adult & Pediatric sarcomas.

Occupational factors such as job type, industry, and exposures to chemicals such as herbicides and chlorophenols have all been found to be suggestive risks for sarcomas. However, no clear consensus exists about the accuracy of these risk factors for sarcoma development, because a good number of completed studies have yielded inconsistent results. Improved study designs with increases in sample size would more clearly define the evidence of these associations. Furthermore, bone development during pubertal growth spurts and history of hernias have also all been found to be associated with sarcoma development. In fact, the majority of studies have consistently found statistically significant associations to exist. These two factors will likely soon become accepted as strongly associated risk factors for sarcoma development once a few additional studies are able to replicate current findings.

Ultimately, drawing strong conclusions can be difficult to make because many of results from these studies have not been adequately replicated. For example, only two studies were retrieved that assessed the potential impact dioxin releasing-industrial incinerators may have on soft tissue sarcoma development [81,82]. However, it is relatively weak to base the foundation of this conclusion on only an ecologic study with 110 cases and a case–control study of 37 cases. Maternal and paternal characteristics such as occupation, age, smoking status, and health conditions experienced during pregnancy are other factors that would also be important for future research to assess. The very limited findings available on these risk factors appear to show significant relationships with sarcoma risk, but these results now require further validation on larger populations. Again, it is difficult to draw any conclusions based on minimally studied and very few replicated significant findings.

The available literature and research on sarcoma risk has shown that these rare diseases are difficult to study. The challenge of studying a rare outcome is that it often requires the assessment of rare exposures. Implementing studies with these characteristics often results in the creation of null or conflicting results. For example, the relationship between occupational factors and the risk of sarcoma development appears to be one of the more common and frequently studied topics [5-10,12,20,21,32,36,45,46,48,59]. However, even with the plethora of papers published on this particular subject matter, clear conclusions can be difficult to draw. Many of the occupational exposure studies lacked the statistical power and adequate sample sizes needed in order to create strong conclusions, thus again, we only can conclude that the identified associations are suggestive. Furthermore, many of these studies were required to group sarcoma subtypes together in order to gain enough statistical power to fully implement their study, which can result in the assumption that many of the sarcoma subtypes have the same etiologic properties.

Future research would benefit from the continued use of advanced record keeping registry systems. Such record systems must span a great deal of time in order to provide adequate sample sizes of rare cancers. Adequate sample sizes would allow for subgroup analyses to be performed separately on the many sarcoma subtypes. Stratified analyses on other potentially associated factors, such as gender, could also be implemented if the sample size permits. Further improvements of the methods utilized in gathering exposure information must also be considered. If a record keeping registry system does not allow for highly accurate exposure information to be obtained, other means of obtaining that information must be employed. It is also important that information obtained on each subject be as comprehensive as possible for adjustment of potential confounders such as socioeconomic status in occupational studies. In addition, we noticed that little is known about the possible biologic mechanisms behind each epidemiologic association among the many relationships assessed in the literature. Future studies of the underlying genetic risks for sarcoma will increase our understanding of the genetic versus environmental contributions to tumorigenesis in this often deadly type of cancer.

## **Competing interests**

The authors declare that they have no competing interests.

## **Authors’ contributions**

ZB conducted the systematic review and drafted the manuscript. MH participated in the systematic review and contributed to drafting of the manuscript. LS & JS conceived the study and participated in the systematic review and contributed to the drafting of the manuscript. All authors read and approved the final manuscript.
